# Case Report: High-concentration Insulin Glargine Overdose Complicated by Hepatic Steatosis

**DOI:** 10.1210/jendso/bvz020

**Published:** 2020-04-22

**Authors:** Ryan Endall, Roland McCallum, John Burgess

**Affiliations:** 1 Department of Diabetes and Endocrinology, Royal Hobart Hospital, Hobart, Australia; 2 School of Medicine, University of Tasmania, Hobart, Australia

**Keywords:** insulin glargine, dextrose, hepatic steatosis, overdose

## Abstract

The use of high-concentration formulations of insulin is becoming more prevalent in the management of patients with diabetes mellitus. Situations of intentional overdose utilizing these agents pose particular challenges because of the altered pharmacology at large doses and the potential complications arising thereof.

A patient with type 1 diabetes mellitus self-administered 4050 units of high-concentration (300 units/mL) insulin glargine, in addition to coingestants. The patient subsequently required 7 days of high-dose dextrose infusion in order to avoid hypoglycemia, with no further insulin needed during this period. The patient also developed reversible hepatic steatosis secondary to the prolonged use of high-dose dextrose.

Owing to the altered pharmacology of high-concentration insulin glargine when administered at large doses in cases of intentional overdose, patients are likely to require a much longer period of supplemental dextrose support than may otherwise be expected when these agents are used at therapeutic doses. The complication of hepatic injury in the form of steatosis also needs to be considered in these patients, and should prompt the use of adaptive prescriptions of intravenous dextrose where possible.

Newer forms of high-concentration long-acting insulin may provide improved glycemic stability to patients with diabetes mellitus. However, the management of intentional overdose of such insulin preparations poses unique challenges. In this report we describe a case of high-concentration (300 units/mL) insulin glargine overdose in the context of type 1 diabetes mellitus (T1DM) that required prolonged high-dependency unit (HDU) level care, and was complicated by the development of dextrose-associated hepatic steatosis.

## 1. Case Presentation

A 45-year-old woman with T1DM presented after self-administering 4050 units of high-concentration insulin glargine (9 1.5-mL pens at 300 units/mL), 300 units of insulin lispro (1 3-mL pen at 100 units/mL), and 40 mg of temazepam and 20 mg of zolpidem, with the intention of suicide. The insulin doses were administered in multiple subcutaneous sites across the abdomen.

Usual management for the patient’s T1DM included continuous subcutaneous insulin infusion (insulin lispro, via a Medtronic® insulin pump device), with an HbA1c 3 months prior of 8.2%. Other medical history included borderline personality disorder and depression, with multiple previous suicide attempts involving subcutaneous insulin, and chronic right foot pain in the context of osteoporosis and previous minimal trauma fractures.

The patient was found by her mother at 07.45 with a reduced conscious state and a blood glucose level (BGL) of 1.1 mmol/L, 5 hours after the overdose; 1 mg of intramuscular glucagon was administered, and the patient’s insulin pump was disconnected. A 15-g bolus of oral glucose was administered when the paramedic team arrived. Following transfer to the emergency department of a tertiary referral center, her conscious state had improved (Glasgow Coma Scale of 15), blood pressure was 122/74 mmHg with heart rate of 76 beats per minute, serum potassium was 3.7 mmol/L, and lowest BGL was 3.8 mmol/L, prompting commencement of a continuous infusion of 10% dextrose at 100 mL/hour, before she was transferred to the HDU at 14.00. The patient denied other coingestants, and a serum paracetamol level was found to be undetectably low (<33 µmol/L).

Management in the HDU consisted of central venous catheter (CVC) insertion, regular monitoring and replacement of serum potassium, and hourly BGL monitoring. The supplemental fluid infusion was escalated to 50% dextrose in response to a BGL of 2.1 mmol/L, and was continued for 144 hours (Table 1). The patient was able to tolerate only minimal oral intake for several days due to reported nausea, before resuming normal diet by day 7. Following cessation of the dextrose infusion, a basal-bolus regimen consisting of multiple subcutaneous insulin injections was commenced. 

**Table 1.** Intravenous dextrose and potassium chloride (KCl) administration during high-dependency unit admission. values in parentheses indicate reference ranges

	Day 1	Day 2	Day 3	Day 4	Day 5	Day 6	Day 7	Total
**Total dextrose (volume)**								
10% dextrose, mL (100 g/L)	1400							1400
50% dextrose, mL (500 g/L)	925	2650	2270	1765	1485	1550	520	11 165
Total glucose, g (weight)	602.5	1325	1135	882.5	742.5	775	260	5702.5
Lowest BGL (mmol/L)	2.1	3.9	1.9	2.9	3.2	3.5	3.0	
Lowest K (mmol/L) (3.5–5.0)	3.2	3.6	3.4	3.8	3.6	3.8	3.4	
Total KCl replacement, mL	250	325	400	50	100	0	50	

**Figure 1.**
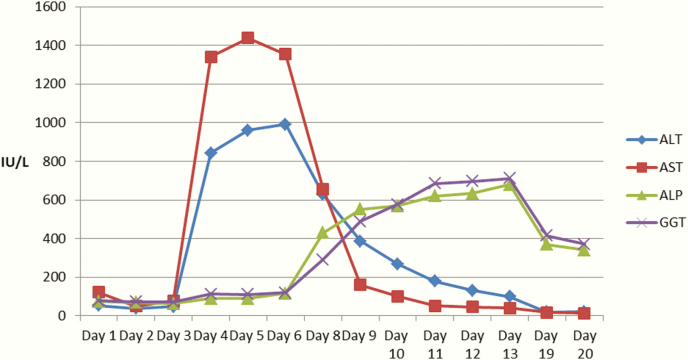
Trends in liver function tests during admission following high-concentration insulin glargine overdose up to day 20. An early rise in alanine aminotransferase (ALA) and aspartate aminotransferase (AST) was noted from day 3, closely matching the rise in bilirubin. A delayed rise in alkaline phosphatase (ALP) and gamnma-glutamyl transferase (GGT) was noted from day 6. No blood tests were taken on day 8.

During the patient’s HDU admission, progressive elevation of aminotransferases (aspartate aminotransferase, AST; and alanine aminotransferase, ALT) was noted, peaking at day 5 (Fig. 1 and Table 2). She remained hemodynamically stable throughout. Regarding other markers of severity for liver disease, bilirubin peaked at 53.9 µmol/L on day 4, international normalized ratio (INR) peaked at 1.3 on day 4, and albumin reached a nadir of 23 g/L on day 10. Liver ultrasound performed on day 9 demonstrated no morphological changes to explain these abnormalities.

**Table 2.** Liver function tests and other markers of hepatic dysfunction. No blood tests were performed on day 7. Values in parentheses indicate reference ranges

	Day 1	Day 2	Day 3	Day 4	Day 5	Day 6	Day 8	Day 9	Day 10	Day 11
AST (IU/L) (<50)	122	50	78	1340	1437	1354	657	161	101	**51**
ALT (IU/L) (<65)	54	39	48	843	960	991	627	387	268	179
GGT (IU/L) (<55)	79	73	71	113	111	121	291	489	577	684
ALP (IU/L) (30–110)	82	69	64	90	89	117	426	551	569	621
Total bilirubin (µmol/L) (<25.0)	4.9	6.0	8.8	57.2	53.9	37.7	22.8	25.1	25.2	20.8
INR (0.9–1.2)		1.2	1.2	1.3	1.3	1.2	1.1			
Albumin (g/L) (35–50)	31	30	30	29	32	28	24	26	23	26

The patient was discharged from HDU to a general ward on day 8 of admission, where she remained an inpatient until day 21 because of mental health issues. AST and ALT levels gradually decreased following cessation of the dextrose infusion, and had normalized by the time of discharge.

## 2.Discussion

This case describes a patient with a background of T1DM who presented following an overdose using a very large dose of high-concentration insulin glargine (4050 units), as well as coingestants as described above. 

The concentrated form of insulin glargine (300 units/mL, Toujeo ®) used in this case is a long-acting basal insulin analogue (1). Insulin glargine differs from human insulin by the addition of two arginines after position B30, and replacement of asparagine with glycine at position A21, which causes the molecule to precipitate when injected into the neutral pH environment of subcutaneous tissue (2). Precipitated drug is then gradually released from this depot. The 300 unit/mL preparation forms a smaller depot than the 100 unit/mL preparation of insulin glargine, resulting in a slower and more prolonged release of insulin, a more stable pharmacodynamic and pharmacokinetic profile, and thus theoretically a reduced risk of hypoglycemia, particularly at night (1, 3). 

Glargine’s duration of effect has previously been demonstrated to vary depending on the dose administered; for example, in a euglycemic glucose clamp study, the duration of metabolic activity was noted to be 24 hours following a dose of 0.3 units/kg, compared with 30 hours following a 0.4 unit/kg dose (4). The variable duration of effect is again demonstrated in this case, with dextrose infusion required for over 144 hours following the initial overdose. Similar findings have also been demonstrated in cases of overdose from other types of insulin, likely to be due to the effect of a larger than usual depot at the injection site, and the downregulation of insulin receptors in target tissues (5, 6). Other patient-specific factors may also influence the duration of hypoglycemia in cases of insulin overdose, including the number of injection sites, coingestants, and reduced clearance or metabolism due to renal failure and hepatic dysfunction, respectively (5).

In this case study, the aminotransferases became progressively more elevated following commencement of the dextrose infusion, and rapidly self-corrected following cessation thereof. Excessive glucose supplementation in the form of dextrose infusion for prolonged periods can lead to hepatic steatosis, as demonstrated in a previous case where a patient received 4040 g of glucose over 72 hours (7). The patient subsequently developed nausea and abdominal pain, and was found to have elevated AST and ALT. De novo lipogenesis in the liver is upregulated by absorption of large quantities of carbohydrate, where the ingested quantity exceeds the body’s immediate needs; insulin also promotes this process (8). Triglycerides are transferred from the liver to the periphery via very low density lipoprotein molecules, whose production depends on apolipoprotein B synthesis; excessive carbohydrate and insulin loading can overwhelm the capacity of apolipoprotein B production to meet demand, thus leading to triglyceride accumulation within hepatocytes, and hepatic steatosis (7, 9). This is the biochemical basis of *foie gras* liver in ducks, with certain breeds being more prone to lipid accumulation in carbohydrate-rich diets (7).

The ingestion of nondisclosed substances as part of the patient’s suicide attempt cannot be fully excluded, and it is possible that such speculative coingestants may have contributed to the pattern of liver function test (LFT) derangement. However, the rapid fall in aminotransferase and bilirubin levels following cessation of the dextrose infusion on day 7 would suggest that dextrose-associated liver injury is a more likely explanation. Previous case reports have not demonstrated the late rise in GGT and ALP which was noted in this case (6, 7).

In the context of massive insulin overdose, adaptive doses of supportive dextrose infusions would seem to be safer than bulk prescription. Consideration of basal glucose utilization, being around 6 mg/kg/min in the presence of large insulin plasma levels (7), is potentially useful. In our patient’s case, a similar approach would have yielded an average hourly dose of 31.50 g (actual dose received was 39.60 g/hour, on average). However, in practice, actual hour-to-hour dosages of dextrose would be influenced by other factors, including real-time BGL results.

A case study published by Fuller et al. (10) outlined a case in which a patient self-administered 4800 of standard-concentration (100 units/mL) insulin glargine as a single subcutaneous injection. On day 4 following the overdose, the insulin depot was surgically excised from the abdominal wall, which was followed by a significant reduction in dextrose requirements and an improvement in LFTs. Such an approach would not be practical in the case discussed here, however, due to the use of multiple subcutaneous injection sites.

Based on a review of the case report literature, this case would appear to involve one of the largest dose insulin glargine overdoses in published history. It demonstrates that, in the context of massive insulin overdose, clinicians should expect hypoglycemia to persist for significantly longer periods of time than the usual pharmacology of insulin at therapeutic doses would suggest. It also serves as a reminder for adaptive dosing of dextrose infusion to avoid the potential complication of hepatic steatosis.

## Additional Information

***Disclosure Summary:*** The authors have nothing to disclose.

***Data Availability:*** The data that support the findings of this study are available from the corresponding author upon reasonable request.

## Abbreviations

AST: aspartate aminotransferase
ALT: alanine aminotransferase
BGL: blood glucose level
CVC: central venous catheter
HDU: high-dependency unit
INR: international normalized ratio
LFT: liver function test
T1DM: type 1 diabetes mellitus
